# Listeria rhombencephalitis in an alcoholic man; A case report and review of literature

**DOI:** 10.1016/j.ensci.2026.100605

**Published:** 2026-02-20

**Authors:** Hussain Al-Sadi, Atanu Basu, Medha Panicker, Bhupal Shrestha, Aimee Crawley

**Affiliations:** a AMU, Glan Clwyd Hospital, Bodelwyddan, UK; b SDEC, Glangwili General Hospital, Carmarthen, UK

**Keywords:** *Listeria monocytogenes*, Rhombencephalitis, Encephalitis, Alcohol withdrawal, Microbiology, Case report

## Abstract

Listeria infections are uncommon in our clinical practice and often overlooked as a differential diagnosis. It is also difficult to uncover due to presentation with a variety of non-specific clinical signs and symptoms, that could mimic other conditions. In our case report, we discuss a rare and challenging case of a middle-aged alcoholic man with Listeriosis affecting the rhombencephalon. He presented with non-specific symptoms, including agitation and confusion, preceded by flu-like illness. These were accompanied by seizures and fever in hospital, as well as intermittently changing neurological deficits. Although his symptoms were put down to alcohol withdrawal initially, the timely identification of *Listeria monocytogenes* in blood cultures could aid to point towards the correct diagnosis and hence prompt a lifesaving treatment. It was later confirmed with MRI imaging of the brain, showing inflammation around the brainstem. Lumbar puncture results were also alluding to a meningoencephalitis picture. Despite the high mortality rate, our patient survived and was discharged from hospital six weeks after the initial admission. We will compare our case with other case reports from the literature. Finally, the discussion will highlight the importance of considering *Listeria* when treating possible encephalitis/ meningitis in patients at risk.

## Highlights

• *Listeria monocytogenes* causes fatal CNS infections, e.g. encephalitis.
• Rhombencephalitis: inflammation of Brainstem and cerebellum (rhombencephalon).
• At-risk group: immune compromised, elderly, neonates, pregnant women, alcoholics.
• Diagnosis is rare, challenging, but serious and life threatening.
• Needs high index of clinical suspicion, cultures, CSF analysis, MRI scan of brain.

## 1 Introduction

*L. monocytogenes*, the only *Listeria* that infects humans, can cause fatal infections of the human central nervous system (CNS), leading to meningitis, micro-abscesses, meningoencephalitis, rhombencephalitis with focal necrosis and hemorrhages. Most fatal consequence are observed in immune-compromised population. However, the non-specific nature of symptoms in healthy immune-competent people makes the diagnosis particularly tricky and easy to miss. In our review, we will highlight the importance of considering meningitis/ encephalitis, even as a differential, when looking after patients with alcohol withdrawal syndrome.

In most healthy individuals Listeria causes self- limiting illness, however, this could be a spectrum ranging from flu-like illness to fatal sepsis. It carries high mortality and morbidity rates. Listeriosis is more common in the immunocompromised, elderly, pregnant and alcoholics. Rhombencephalitis, mostly caused by L. *monocytogenes*, refers to inflammation of the hind brain (Brain stem and cerebellum).

## 2 Case report

A male patient in his 50s, who was quite independent, was brought to the hospital by ambulance, after being found by a family member on the floor having seizures. As per documentation from paramedics, patient was confused and had soiled himself. He was also febrile with temperature recorded 38.8C.

A collateral history from the family suggests that patient had been complaining of flu like prodromal illness, dry cough, lethargy, body aches, abdominal pains, and diarrhea for the last 3 days.

When the patient arrived in A&E, he was found confused, agitated, combative and restless. Sweating was also noted, with mild tremor in his upper limbs. He had also vomited once.

Upon assessment by the medical team, he was found sweaty, clammy and tachycardic at 127 bpm. He was tachypneic with respiratory rate reaching 40/min, and hypoxic with saturations of 92% on room air. His heart sounds were normal. His chest was clear, and abdomen was soft non distended but generally tender. Although neurological examination was limited due to agitation and poor compliance, there were no obvious focal neurological deficit. Extraocular movements and cranial nerves seemed intact. No neck stiffness was appreciated. Patient's Glasgow Coma Scale was 14/15 but he kept complaining of headaches throughout the day. No skin rashes, or signs of anaemia/ jaundice were noted.

### 2.1 Past medical history

includes Gastroesophageal reflux disease, Fatty Liver, Alcohol misuse, Previous use of Psychedelics, Depression. Past surgical history: Ischiorectal abscesses and Anal fistulas previously drained with seton insertion, Scrotal abscess with communicating anal fistula drained.

### 2.2 Social history

Patient was independent, heavy smoker, long history of alcohol misuse (last drink was thought to be 1 or 2 nights before). No recent travel, no dietary changes. Current medications: Thiamine, Omeprazole, Sertraline.

Basic blood tests showed neutrophilic leukocytosis (WBC: 26 × 10^9/L [Normal: 4.0–11.0]. Neut: 20 × 10^9/L [Normal: 1.7–7.5]). Mild hyponatremia and hypokalemia (Na: 131 mmol/L. K: 3.0 mmol/L).

Urea: 2.8 mmol/L [Normal: 2.5–7.8]. Creatinine: 101 umol/L [Normal:58–110]. (Baseline Creatinine: 57). Elevated liver enzymes: Alanine transaminase (ALT): 237 U/L [Normal: <41]. Alkaline phosphatase (ALP): 147 U/L [Normal: 30–130].

Initial C-reactive protein (CRP): 21 mg/L [Normal: <5]. CRP was 149 mg/L the following day. Procalcitonin: 13.2 μg/L [Normal: <0.05].

Creatine kinase (CK) was also high at 7275 U/L [Normal: 40–320]. Serum lactate was raised at 4.2 mmol/L [Normal: 0.5–1.6].

An electrocardiogram (ECG) showed sinus tachycardia at 133 bpm. Unenhanced CT scan of the head was reported as normal. Chest X-ray was unremarkable.

Patient was started on IV Fluids with Potassium, IV Tazocin (Piperacillin / Tazobactam) as broad-spectrum antibiotic for sepsis of uncertain origin and IV Pabrinex. The doctors also asked to commence patient on Alcohol Withdrawal Assessment tool as per local guidelines, ‘PRN’ Librium (Chlordiazepoxide) for *Clinical Institute Withdrawal Assessment – Alcohol, revised (CIWA Ar)*.

Due to ongoing fever, throat swab was sent to test for extended panel of respiratory pathogens: negative results, including Covid 19. Blood and Urine cultures were also taken.

Couple of days following admission, the clinical picture and level of consciousness changed. On repeat neurological examination, gaze evoked nystagmus was noted bilaterally. This was put down to alcohol withdrawal and Wernicke's Encephalopathy at the time. The nursing staff reported patient having visual hallucinations. Patient's GCS started to fluctuate, dropping to 9/15 at times. He became more drowsy, weak, and intermittently unresponsive, compared to very restless and agitated on admission. Intensive Care assessment was done but not transferred to ITU at that stage.

After several spikes of high temperature, Blood cultures grew Gram positive bacilli. *Listeria monocytogenes* cultured in both bottles. Microbiology department advised changing antibiotics to high dose Amoxicillin 2 g six times daily, and Gentamicin.

Lumbar puncture (LP) procedure was delayed initially due to agitation and low GCS. However, LP was done later. Cerebrospinal fluid (CSF) was yellow in colour, slightly turbid. Analysis revealed; Pleocytosis. WBC: 269 × 10^6/L. Lymphocyte 100%. In the Gram stain: No organisms were seen. Culture of CSF: No growth. CSF protein was raised: 3.61 g/L [Normal: 0.15–0.45]. CSF glucose was: 2.3 mmol/L [Normal: 2.2–3.9]. Polymerase chain reaction (PCR) for Herpes simplex 1 & 2, Meningococcal, Pneumococcal, and Enterovirus was negative. Hepatitis screen and HIV test were negative.

**Fig. 1:**
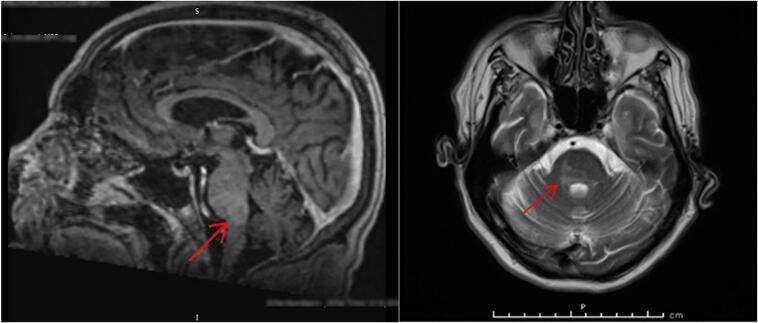
Magnetic resonance imaging (MRI) of the patient.

Magnetic resonance imaging (MRI) of the head showed possible mild enhancement around the brainstem. Fig. 1 Microbiology department advised this is typical report for *Listeria* rhombencephalitis, inflammation around the rhombencephalon (hindbrain: brainstem and cerebellum).

Further daily neurological examination showed that patient was developing pathological reflexes and evolved into quadriparesis, all his limbs were weak and stiff. Babinski sign was positive on the right side. Speech was noted to be slurred. No cerebellar signs were appreciated.

Over the course of the next two weeks, clinical condition was fluctuating with issues related to immobilization, poor oral intake and low potassium. Patient was developing edema due to low albumin, also developing ileus likely due to immobility and low potassium level, which was being replaced intravenously all the time. Patient was also given IV Albumin 20% daily.

Further, investigations like fungal screen were negative. Echocardiogram was done and did not show evidence of infective endocarditis. Due to deranged Liver enzymes, ultrasound scan of the liver was performed; it revealed fatty changes of the liver. IV Meropenem 2 g, three times daily was initiated by microbiology department.

In the following few weeks, patient showed significant improvement in GCS, and slow improvement in neurological deficits. With help of physiotherapy, he was able to start walking with a Zimmer frame, with only mild residual weakness of the right arm, and was ready for discharge. Our patient survived despite his poor prognosis and unfavourable prognostic features. He was discharged from hospital after nearly six weeks of admission.

## 3 Differential diagnosis

In our case, reaching the diagnosis was initially challenging. The presenting symptoms changed a lot during the first few days of admission. Given the patient was known alcoholic, the presenting seizures at home were treated as possible alcohol withdrawal seizures, especially knowing the patient's last alcohol drink was 1–2 nights before admission.

During the first couple of days, patient was agitated, sweating, confused and restless, to conclude the picture of alcohol withdrawal syndrome. Features of confusion and nystagmus later was thought to be part of Wernicke's Encephalopathy related to chronic alcohol misuse, indicating IV Pabrinex. The high temperature spikes were put down to possible Delirium Tremens as a first thought. The unstable vitals were however, pointing towards sepsis of unknown source. Fluctuating/ drop of GCS was also initially difficult to explain. One possible differential here was overmedication of Librium (Chlordiazepoxide) & Haloperidol. Low GCS with deranged liver enzymes were also thought to be caused by Hepatic encephalopathy, however a normal Ammonia level ruled that out. The headaches with fever spikes did point towards considering the diagnosis of encephalitis or meningoencephalitis.

Inflammation of the brainstem can have wide variety of causes; demyelination disorder (e.g. multiple sclerosis) or autoimmune disease (e.g. Behçet's) or even part of paraneoplastic syndrome. Rarely, it can be caused by neoplastic diseases, like lymphoma. Infections could include enterovirus or herpesviruses. [1] Bacterial aetiologies, like tuberculosis should also be considered in certain geographical areas. In patients with severe immune deficiency, JC virus infection causing Progressive multifocal leukoencephalopathy (PML) should be in the differential, although rare. [1]

## 4 Further discussion

*Listeria monocytogenes* is facultative intracellular, Gram-positive rods. It is motile and non-spore-forming. It has multiple virulence factors that make it resistant to eradication processes. [1] [2].

*L. monocytogenes* resides in soil, water, and decayed substances. It has well adapted to live in both, the soil and inside eukaryotic host cells. [3]

*Monocytogenes* is the only strain of Listeria that infects humans. It is a food borne pathogen; up to 99% of infections happen via the oral route, from contaminated foods, like unpasteurized dairy products, fresh soft cheese, raw vegetables, salads, coleslaw, or even undercooked meat. [2] [4] Transmission to neonates occurs via vertical transmission. *L. monocytogenes* is responsible for several food­borne epidemic diseases around the world and has high associated mortality rates. [1]

In the healthy population, Listeria infection is usually limited to mild febrile gastroenteritis causing diarrhea, with abdominal pain, myalgias, and headaches. This group usually recovers in few days, without the need for antibiotics therapy. However, in individuals at risk, such as immunocompromised, the bacterium can cause fatal disseminated infections, leading to meningitis, encephalitis, rhombencephalitis or brain abscesses and sepsis. [4] Among the bacteria that can infect the CNS, *L. monocytogenes* is one of the deadliest known. For the record, Listeria is thought to be the third most common cause for bacterial meningitis. [2] [5].

Rhombencephalitis refers to inflammation of hindbrain. It has variety of clinical presentations and carries a very high mortality and morbidity rates. It can be caused by infections, autoimmune process, or part of paraneoplastic syndrome. The most common infective cause is L. *monocytogenes*, as in our case. Clinical features include fever, neck stiffness, headache, vomiting, change in GCS, seizures, tremor, ataxia, changing focal neurology, and pathological reflexes. Involvement of long tracts and pyramidal tracts can lead to hemi/ quadriparesis, or hypesthesia, etc. It's worth mentioning that meningeal signs are often absent. Involvement of cerebellum could give features of ataxia, or dysmetria. Furthermore, cranial nerves can be involved as well causing asymmetric palsies which are common and point towards brainstem involvement. [1] [4] Respiratory failure caused by damage to the medulla may manifest early and require invasive ventilation support in ITU. It can happen in up to 40% of patients with rhombencephalitis. [2] Finally, encephalopathy, coma and death resulting from increased intracranial pressure, affecting the brainstem, can progress quickly and is fatal unless diagnosed and tackled early.

In CNS Listeriosis, brainstem involvement occurs in up to 11–20% of the cases [1], compared to meningeal involvement which may happen in 48% of all Listeria infections. [2]

People at risk of Listeria infection include elderly, immunocompromised, alcoholics, pregnant women, and neonates. [2] However, rhombencephalitis can also affect immune-competent adults. [1]

CSF analysis in *Listeria* rhombencephalitis is not always typical of a bacterial infection and can be mistaken for viral causes. It can even be completely normal, making the diagnosis challenging. [1] Relatively normal or low normal CSF glucose was observed. Moderate pleocytosis with lymphocyte predominance, despite of peripheral blood neutrophilic leucocytosis, was noted too. Most literature noted elevated proteins level. [2] [6] In some reported cases, neutrophil-predominant pleocytosis was mentioned. [1] It was concluded in few other case reports, that pleocytosis could be neutrophilic or lymphocytic. [2] [9] Blood cultures are more likely to be positive than CSF cultures.

MRI with contrast is superior to CT scans when diagnosing rhombencephalitis, hence it's the modality of choice. [1] [6] Rhombencephalitis is characterized by cerebellar and/or brainstem enhancing lesions, which can be ring, nodular, or even abscess shape ischemic or hemorrhagic lesions. [2] [8].

When it comes to treatment, it's important to mention that L. *monocytogenes* is resistant to cephalosporins, such as Ceftriaxone, which is usually started as empirical therapy for meningitis as per our local guidelines. [1] [7] Ampicillin, Amoxicillin, or Penicillin G are the best choice of antibiotics recommended. Meropenem has also been used. According to some resources, combination with Gentamicin adds synergistic effect with Penicillins and may be given to patients with immune impairment. [1] [7] In addition, Rifampin has good bacteriostatic effects against L. *monocytogenes*, as well as the ability to cross the blood brain barrier. [9] Co-trimoxazole is second line, in case of penicillin allergy. [1] [7] Treatment duration recommended: 3–6 weeks of IV antibiotics. [2] [9]

Further, it's important to discuss the management of seizures caused by intracranial edema and increased intracranial pressure which is associated with higher morbidity and mortality. The use of antiepileptic drugs prophylactically needs more large studies to see effects. There is no available current evidence to support their use.

Keeping an eye on serum Na level is of paramount importance, particularly looking for complications such as SIADH and cerebral salt wasting syndrome. In addition, watching for signs of increased intracranial pressure.

Regarding Steroids, there is no sufficient evidence in literature to support the use. [9]

Mortality and morbidity rates are high, particularly due to delayed recognition and treatment, as well as drug resistant strains. Mortality can reach 35% in rhombencephalitis, with residual neurological symptoms remaining in 55% of survivors. [2]

## 5 Conclusion

Rhombencephalitis is a rare but possibly fatal type of encephalitis that affects the hindbrain. It can present with non-specific signs, and wide range of neurological symptoms, hence can easily be misdiagnosed. It needs quick and early recognition to prevent severe neurological sequelae and severe sepsis. It should be considered especially in patients at risk even when cultures are negative.

## 6 Review of literature

In Table 1, we review similar *Listeria* CNS infections reported in literature. We note different presentations, risk factors, and course of disease.

**Table 1:** Review of Literature.

Publication	Age & Gender	Signs & Symptoms (brief description)	Risk factors	Imaging	CSF analysis	Antibiotic treatment used	Outcome
Liming Cao 2021. [10]	50 Years, male	Headache, dizziness, malaise, vomiting, facial weakness. Patient was alert initially. Later, patient had fever, neck stiffness, somnolence, dysphagia, slurred speech. Respiratory failure.	Nil	Initial CT head: Normal. Day 4: MRI head: multiple foci on brainstem.	CSF: WBC: 835 × 10^6/L. Glucose: 3.0 mmol/L. Protein: 2.19 g/L. No organism in culture. *L. monocytogenes* in blood culture.	Initially Ceftriaxone & Ganciclovir, + mannitol. Later: ampicillin, amikacin, and meropenem.	Treated in intensive care unit (ICU), initiated. After 2 months, was discharged. Able to walk but had respiratory center damage
P Fredericks 2015. [1]	59 years, female	1­week history of flu like illness, ataxia, Facial sensory disturbance, hoarseness of voice. Fever, frontal headache. Dropped level of consciousness. Coma and bilateral papilledema	Not mentioned	MRI brain: signal abnormalities & enhancement in pons, middle cerebellar peduncle and basal ganglia.	CSF protein and glucose levels: Normal. WBC: 4 cells per high power field (polymorphs). No growth. Blood culture on day 15: revealed L. *monocytogenes*.	Telithromycin & Moxifloxacin. IV methylprednisolone and Aciclovir.	Patient passed away after 3 weeks. Microabscesses confirmed in autopsy
P Fredericks 2015. [1]	51 years, male	5 days history of headache, nausea and vomiting. Ataxia. Left facial numbness, Right side Horner's, diplopia, Nystagmus, CN VI palsy, Left side dysmetria.	Alcoholic. HIV + ve	CT head: ring lesions in dorsal Pons & left middle cerebellar peduncle.	CSF Protein: raised at 0.69 g/L. Glucose: 3.2 mmol/L. WBC: Raised: 93 per high power field, mostly polymorphs. Cultures of CSF and blood were negative.	Rifampicin, Isoniazid, Pyrazinamide, Ethambutol. Ampicillin from day 11.	After 33 days. Patient alive, with residual facial numbness & gait ataxia.
Thomas R. 2021. [2]	47 years, male	Headache, vomiting for 1 week. Fever, neck ache, confusion. Diplopia and ataxic gait. Hiccups, +ve Kernig and Brudzinski. Later, confusion, LMN facial palsy, horizontal Nystagmus. Impaired eye abduction (CN VI). Ankle weakness. Left cerebellar signs. Later, b/l limb weakness.	Nil	MRI brain: dorsal Pons hyperintensity. Bilateral cerebral & cerebellum foci indicating acute hemorrhages.	CSF WBC: 131 × 10^6/L, 90% Lymphocytes. Protein: 3 g/L. Glucose: 0.2 mmol/L. CSF culture +ve L. *monocytogenes*	Initially Ceftriaxone & Vancomycin. Then changed to Ampicillin & Gentamicin for 6 weeks. Levofloxacin added to consider bacterial resistance.	Had shallow breathing, hence intubated. After six weeks, patient was discharged, able to walk. Residual ophthalmoplegia
Aslıhan Yerlikaya, 2019. [9]	65 years, female	3 days history of headache & fever. Later, +ve Brudzinski & Kernig. Less responsive and drop in GCS. Later, Muscle power reduced to 3/5 particularly lower limbs. +ve Babinski. Impairment of lateral eye movement, diplopia, papilledema. Then hypotonia & pathologic reflexes. CN VI palsy, Nystagmus, increased ICP, quadriparesis. Also developed seizures.	Cervical Ca. On Chemotherapy. History of **cheese** consumption	MRI head: leptomeningeal enhancement. Repeat MRI: intracranial edema & hydrocephalus	CSF glucose: 2.8 mmol/L. High Protein: 2.39 g/L. WBC: 392 × 10^6/L, mostly neutrophils 86%. CSF culture: +ve L. *monocytogenes*	Initially Meropenem, then added Vancomycin. Mannitol used to reduce ICP. After CSF culture: Ampicillin + Gentamicin started for 6 weeks. Also, Rifampicin added.	Intubated and ventilated in ICU. Not mentioned if patient survived.
Christopher Thomas, 2018. [11]	79 years, male	Confusion, headache. Later: less responsive, GCS drop to 12/15. Horizontal and vertical nystagmus. Bilateral dysmetria. Reduced power (3/5) in right upper and lower libs, and right-side brisk reflexes.	Prostate cancer (receiving hormonal therapy)	Initial CT head: Normal. Later, MRI head: ring enhancing lesions in brainstem and cerebellum	CSF protein: 1.15 g/L. Glucose: 3 mmol/L CSF WBC: 63 × 10^6/L, 90% polymorphs. CSF culture: negative. ---- BC: +ve for L. *monocytogenes*	Initially Ceftriaxone and Aciclovir. Changed to Amoxicillin & Co-trimoxazole after BC + ve for 6 weeks. Then further 6 weeks of Co-trimoxazole after discharge.	Initially required intubation & ventilation during admission. Discharged after 6 weeks. Reviewed 2 months later: had residual nystagmus, and dysmetria.
Christopher Thomas, 2018. [11]	66 years, female	Two days history of diplopia, unsteadiness. Gen unwell for 10 days. Headache and lethargy. Right side nystagmus and dysmetria, Ataxic gait. Right side brisk reflexes and + ve Babinski.	Nil	CT head: normal. MRI head: hyperintensities of cerebellum, brainstem, and internal capsule.	CSF protein: 1.67 g/L. Glucose: 3.6 mmol/L. CSF WBC: 195 × 10^6/L, (95% polymorphs) CSF culture: negative ----- BC: negative.	Initially Ceftriaxone & Aciclovir. Later added Amoxicillin & Co-trimoxazole for 6 weeks.	Resolution of MRI findings. Recovery of the neurological deficits. Discharged with 6 weeks of Co-trimoxazole.
Piotr Czupryna 2014. [12]	61 years, female	Two weeks history of headache, vertigo, nausea, drowsiness. Fever. GCS 12/15. Neck stiffness. – Later: GCS 10/15 and less responsive. Later unconscious.	?? Recent tick bite.	2× CT head: Normal. MRI head: inflammatory lesions, irregular hyper-intense lesions in pallidum, internal capsules, and right cerebellum.	CSF Pleocytosis: WBC: 49 × 10^6/L (Lymphocyte predominance). Protein: 0.67 g/L --- BC: ampicillin-resistant L. *monocytogenes,* sensitive to meropenem.	Initially Ceftriaxone, with Mannitol. Aciclovir and Dexamethasone were given as well.	Transferred to ICU for ventilation and inotrope support. Remained in Vegetative state. Later passed away.
Alison E. P, 2022. [13]	31 years, female. 21 weeks pregnant	Fever, frontal headache. Initial neurology: unremarkable. Myalgia for 8 days?? Viral illness. Day 13: Patient confused & drowsy. Seizures. Brisk reflexes. Day 15: GCS 10/15. Eye movement impairment. R side mouth drooling. Bilateral CN VI palsy. Clonus & hyperreflexia.	Pregnant	MRI without contrast: normal. MRI day 17: hyperintensities in basal ganglia, brainstem, & dentate nuclei.	CSF WBC: 75 × 10^6/L (75% Lymphocytes) Protein: 1.88 g/L. Glucose 2.1 mmol/L. No organism in culture.	Initially empirical Ceftriaxone & Metronidazole (local guide for PUO). Later, Amoxicillin & Aciclovir added to cover intracranial infections. Later, switched to Meropenem and Gentamicin added. ** Levetiracetam used for seizures.	ICU management. Discharged after 42 days. Had residual broad based gait. Mild limb ataxia + intentional tremor. Delivered at 39 weeks (C-section) Neonate was healthy.

## CRediT authorship contribution statement

**Hussain Al-Sadi:** Writing – original draft, Resources, Formal analysis, Data curation. **Atanu Basu:** Writing – review & editing, Supervision. **Medha Panicker:** Validation, Supervision, Project administration, Methodology, Investigation, Formal analysis, Conceptualization. **Bhupal Shrestha:** Writing – review & editing, Visualization. **Aimee Crawley:** Writing – review & editing, Visualization, Software.

## Funding

Writing this case report did not receive funding aid of any form.

## Declaration of competing interest

The authors declare that they have no known competing financial interests or personal relationships that could have appeared to influence the work reported in this paper.
